# Multivariate analysis of covariates of adherence among HIV-positive mothers with low viral suppression

**DOI:** 10.1186/s12981-018-0197-8

**Published:** 2018-03-31

**Authors:** Tamara Nsubuga-Nyombi, Simon Sensalire, Esther Karamagi, Judith Aloyo, John Byabagambi, Mirwais Rahimzai, Linda Kisaakye Nabitaka, Jacqueline Calnan

**Affiliations:** 1 USAID Applying Science To Strengthen and Improve Systems (ASSIST) Project, USAID, University Research Co., LLC, Plot 40, Ntinda II Road, P.O. Box 28745, Kampala, Uganda; 2 Ministry of Health, Kampala, Uganda; 3 United States Agency for International Development (USAID), Kampala, Uganda

**Keywords:** Low viral suppression, Retention, Mothers, ART

## Abstract

**Background:**

As part of efforts to improve the prevention of mother-to-child transmission in Northern Uganda, we explored reasons for poor viral suppression among 122 pregnant and lactating women who were in care, received viral load tests, but had not achieved viral suppression and had more than 1000 copies/mL. Understanding the patient factors associated with low viral suppression was of interest to the Ministry of Health to guide the development of tools and interventions to achieve viral suppression for pregnant and lactating women newly initiating on ART as well as those on ART with unsuppressed viral load.

**Methods:**

A facility-based cross-sectional and mixed methods study design was used, with retrospective medical record review. We assessed 122 HIV-positive mothers with known low viral suppression across 31 health facilities in Northern Uganda. Adjusted odds ratios were used to determine the covariates of adherence among HIV positive mothers using logistic regression. A study among health care providers shed further light on predictors of low viral suppression and a history of low early retention. This study was part of a larger national evaluation of the performance of integrated care services for mothers.

**Results:**

Adherence defined as taking antiretroviral medications correctly everyday was low at 67.2%. The covariates of low adherence are: taking other medications in addition to ART, missed appointments in the past 6 months, experienced violence in the past 6 months, and faces obstacles to treatment. Mothers who were experiencing each of these covariates were less likely to adhere to treatment. These covariates were triangulated with perspectives of health providers as covariates of low adherence and included: long distances to health facility, missed appointments, running out of pills, sharing antiretroviral drugs, violence, and social lifestyles such as multiple sexual partners coupled with non-disclosure to partners. Inadequate counseling, stigma, and lack of client identity are the frontline factors accounting for the early loss of mothers from care.

**Conclusions:**

Adherence of 67% was low for reliable viral suppression and accounts for the low viral suppression among HIV-positive mothers studied, in absence of any other factors. This study provided insights into the covariates for low adherence to ART and low viral load suppression; these covariates included taking other medications in addition to ART, missed appointments in the past 6 months, feels like giving up, doesn’t have someone with whom to share private concerns, experienced violence in the past 6 months, and faces obstacles to treatment and confirmed by health providers. To improve adherence, we recommend use of a screening tool to identify mothers with any of these covariates so that more intensive adherence support can be provided to these mothers.

## Background

Human immunodeficiency virus (HIV) causes a chronic infection that leads to a progressive disease. Without treatment, most persons with HIV develop acquired immunodeficiency syndrome (AIDS) within 10 years of infection, which results in HIV transmission to sex partners, substantial morbidity, and eventually death [1]. Testing identifies infected persons and is the entry point to a continuum of HIV health care and social services that improve health outcomes, including survival. This continuum includes diagnosis (HIV testing), linkage to and retention in continuous medical care for HIV, prevention counseling and other services that reduce transmission, and consistent antiretroviral therapy (ART) for viral suppression [1].

While the ultimate goal of ART is to reduce HIV-related morbidity and mortality, the initial goal is full and durable viral suppression. Full viral suppression allows for utmost reconstitution or maintenance of immune function and minimizes the emergence of drug-resistant viruses caused by ongoing replication in the presence of antiretroviral drugs. The current guidelines in Uganda define viral suppression as reducing the function and replication of the HIV virus to fewer than 1000 copies/mL [2]. In addition to reducing HIV-associated morbidity and mortality for the individual patient, viral suppression is an important goal for epidemic control at the population level, since when viral suppression is achieved and maintained, HIV transmission is substantially decreased [2].

In general, people with HIV need to use a combined antiretroviral therapy also known as highly active antiretroviral therapy (HAART) to achieve long-term viral suppression. Combination ART is necessary because HIV can mutate when a single drug (also referred to as monotherapy) is used. Sometimes, a particular ART regimen cannot help an HIV-positive patient achieve an undetectable viral load. In such cases, new combinations of drugs need to be tried until full viral suppression is achieved [3].

The World Health Organization recommends viral load testing as the preferred method for monitoring the clinical response to ART of people living with HIV infection (PLHIV). Viral load monitoring of patients on ART helps ensure early identification and confirmation of ART failure and enables clinicians to take an appropriate course of action for patient management.

While the ultimate goal of ART is to reduce HIV-related morbidity and mortality, the initial goal is full and durable viral suppression. Long-term viral suppression requires near-perfect adherence to antiretroviral medications [3]. Other factors include genetic differences in drug metabolism, severe baseline immune suppression, prior drug resistance, and concurrent opportunistic infections. In practice, highly consistent adherence requires a patient on a twice-daily regimen not to miss or substantially delay more than three doses of antiretroviral medications per month [4] and as few as one dose per month if the regimen consists of a once-daily backbone drug. This degree of adherence is far greater than that commonly associated with other chronic diseases and is quite difficult for most patients to maintain over the course of a lifelong illness [4].

In Uganda, despite being an early success story in the reduction of HIV infection rates, there are myriad challenges in the recent era of accelerated ART scale-up, including low viral suppression among PLHIV on antiretroviral drugs, with adherence being a major predictor. Ongoing vulnerabilities of poverty, famine, inadequate monitoring of clients on ART, stigma, and violence compound treatment-related costs and concerns [5].

With funding support from the U.S. President’s Emergency Plan for AIDS Relief (PEPFAR), the United States Agency for International Development (USAID) Applying Science to Strengthen and Improve Systems (ASSIST) Project supported interventions for the elimination and prevention of mother-to-child transmission of HIV (PMTCT) through quality improvement activities in PMTCT implementing sites in Northern Uganda. Under this initiative, Ministry of Health (MoH) PMTCT sites were supported to monitor viral load in HIV-positive mothers on ART, retain these mothers in care, and maintain adherence to treatment in order to reduce the risk of transmission to their HIV-exposed babies. Facility teams identified mothers who were eligible for viral load testing and to ensure follow-up for those with low viral suppression. Health workers were trained to interpret viral load results and provide intensive adherence counselling for mothers that were not suppressing. Community-based linkage facilitators were also trained to provide health education for adherence and follow up mothers in the community so as to keep them in care. Linkage facilitators also identified mothers who give birth in the community and referred them for HIV testing so as to initiate those positive on ART.

In December 2016, results generated from the MoH central public health laboratory showed that 217 pregnant and lactating women receiving care in health facilities in 15 districts of Northern Uganda had not achieved viral suppression and had viral load values of more than 1000 copies/mL. The present study was carried out to gain further insight into the reasons for non-viral load suppression amongst HIV-positive pregnant and lactating women in Northern Uganda in order to inform improvement interventions to increase viral load suppression in this particularly vulnerable population. Adherence in this study was operationally defined to mean taking antiretroviral medication correctly every day.

## Methods

### Study subjects

The study was conducted among 122 HIV-positive pregnant and lactating mothers known to have low or no viral suppression based on the results of their most recent viral load test results. The current guidelines in Uganda define viral suppression as reducing the function and replication of the HIV virus to fewer than 1000 copies/mL. Mothers who had not attained viral suppression as per these guidelines were therefore considered to have low or no viral suppression for purposes of this study. Study participants were identified from 13 health facilities in 15 districts of Northern Uganda where the USAID ASSIST Project was supporting PMTCT services. All HIV-positive pregnant women known in these 13 facilities to have low viral suppression and were present at the study sites at the time of the study were included. Additional information was collected through focus group discussions with health care providers and linkage facilitators connected to these 13 facilities, to understand their perceptions of the underlying causes of low viral load suppression, non-adherence, and low early retention among their clients.

### Study design

The evaluation was a facility-based, cross-sectional study with retrospective medical record review for the study subjects for whom medical records could be located. The study utilized a mixed methods approach to enrich information to inform improvement strategies and the development of tools to address the covariates of low adherence responsible for low viral suppression. Cross-sectional data was collected through interviews with the identified HIV-positive mothers with low viral suppression on factors identified from the literature underlying low early retention in care. Focus group discussions were conducted with service providers to obtain their perspectives on predictors of low viral suppression. Retrospective data was collected for 68 of the 122 subjects on recorded adherence at the time of taking the viral load sample, clinical failure in the past 12 months, and immunological failure in the past 12 months. This constituted the sample whose records could be located at the facility at the time of the study.

### Data collection

The structured interview questionnaire asked about mothers’ socio-economic and demographic characteristics, HIV disclosure, social support and isolation, stigma and discrimination, treatment with ARVs and other drugs including traditional medicine, adherence to treatment, length of treatment, exposure to violence, and satisfaction with the adequacy of ART preparation counseling.

Focus group discussions (FGDs) with health care providers and linkage facilitators in the care of PLHIV obtained information on the category of mothers who have low viral suppression, the underlying causes of low viral suppression and low early retention, adherence to treatment, reasons for non-adherence, and suggestions on addressing low viral suppression, non-adherence, and low early retention within the facility context. A total of eight FGDs were conducted and comprised of both male and female health care providers and community linkage facilitators from different level facilities. Retrospective data was extracted from patient files and covered clinical assessments for clinical failure, immunological failure, treatment regimen, and adherence at the time of taking last viral load test.

### Analysis

Quantitative data was analyzed using SPSS 16 and comprised of participant interviews and clinical records data. Univariate analysis was conducted to describe clinical records data related with adherence. Multivariate analysis was carried out to estimate covariates of adherence among HIV-positive mothers with low viral suppression. To assess the effect sizes of covariates of adherence, we analyzed data from the participant interviews using logistic regression model. The effect size of the covariates and strength of association was depicted using the adjusted odds ratios (AOR). Necessary model diagnostics and exploratory data analysis were checked at the respective stages of the analysis.

Focus group discussions with health care providers were transcribed verbatim into full text and coded using a thematic analysis. The quotations included in the text best represented the range of ideas voiced around key themes. The quotations were carefully edited without altering the meaning. To maintain anonymity, these quotes are identified by place of discussion only.

### Ethical considerations

This study was reviewed and approved in accordance with the guidelines for conducting research and was part of a wider study of mothers under integrated care. It was reviewed by an approved local Institutional Review Board, and the Uganda National Council of Science and Technology. Administrative clearances were obtained from district health management teams and from the community leaders within the intervention areas. Participation was entirely voluntary, and consent was based on full information about the study and intervention.

## Results

### Profile of HIV-positive mothers

The majority (78%) of the HIV-positive mothers interviewed were aged 25–34. They were largely illiterate with primary education (66.4%). More than half of them (54.1%) traveled 6 km or more to the health facility for treatment. Of these, 38.5% travelled as far as between 10 and 20 km to the health facility and spent at least 1.6 US dollars on transport alone for each visit to the facility. Almost all of these women lived with someone aged above 5 years (98%). They were predominantly married (63.9%). However, 23% of all mothers had non-regular partner in the past 12 months preceding the study. This was corroborated by health providers and linkage facilitators, who indicated that the majority of these mothers were in unstable relationships and/or multiple sexual relationships. More than half (51.6%) of mothers had experienced some form of violence in the last 6 months and 16% take alcohol. These results are contained in Table 1.

**Table 1:** Characteristics of HIV-positive mothers

Socio-demographic information	Number (n = 22)	Percentage
Age		
15–24	26	21.3
25–29	28	23.0
30–34	67	54.9
45+	1	0.8
Level of education		
None	31	25.4
Primary	81	66.4
O level	10	8.2
Marital status		
Married	78	63.9
Single	10	8.2
Divorced/separated	22	18.0
Widowed	12	9.8
Has non-regular partner	28	23
Distance from home to the health facility		
Less than a km	10	8.2
2–3 km	19	15.6
4–5 km	27	22.1
6–10 km	19	15.6
More than 10 km	47	38.5
Cost of each visit to the facility		
Less than $0.2	2	1.6
Between $0.2 and $0.6	19	15.6
$0.8–$1.4	23	18.9
$1.6–$2.7	34	27.9
More than $2.7	30	24.6
Walks to the facility	14	11.5
Lives with other people in household		
Lives alone	3	2.5
Lives with other people above 5 years	199	97.5
Victim of violence in last 6 months		
Yes	63	51.6
No	59	48.4
Takes alcohol		
Yes	20	16.4
No	102	83.6

Table 2 provides information on use of ART and traditional medicine among mothers with low viral suppression. Results in Table 2 show that only 67% of the mothers were taking ARVs daily and about 19% were taking ARVs along with other medicines, including traditional medicine/local herbs (8.2%). Close to a quarter of the mothers with low viral suppression had changed ARVs since starting on treatment and 18% of all mothers had been admitted in the hospital in the past 1 year preceding the study.

**Table 2:** Consistence in the use of ARVs and traditional medicine

Medication	Number (n = 122)	Percentage
Frequency with which ARVs are taken		
Everyday	82	67.2
Not every day	40	32.8
Taking other medication with ARVs		
Yes	23	18.9
No	99	81.1
Takes traditional medicine and ARVs		
Yes	10	8.2
No	112	91.8
Changed ARVs since starting ART		
Yes	29	23.8
No	93	76.2
Fall out of treatment 3 months after initiation on ART		
Yes	27	22.1
No	95	77.9
Admitted in hospital in the past 1 year		
Yes	22	18.0
No	100	82.0

The study investigated early retention of mothers in care so as to determine its effect on low viral suppression. Early retention was operationally defined to mean retention in care for the first 3 months after enrollment for ART. We found out that only 22.1% (n = 27) of the mothers studied had dropped out of treatment with 3 months after initiation on ART.

### Clinical characteristics of the study participants

During the assessment, we extracted clinical data from patient records for only 68 of the 122 mothers due to documentation gaps. The findings are contained in Table 3. We found that 41% of the mothers out of the 68 whose clinical data was extracted, were on tenofovir, lamivudine, and efavirenz (TDF–3TC–EFV) at the time of taking the last viral load measurement.

**Table 3:** Clinical characteristics of study participants

Clinical information	Frequency	Percent
Patient regimen at the time of taking last viral load	n = 68	
AZT/3TC/NVP	15	12.3
TDF/3TC/EFV	50	41.0
TDF/3TC/NVP	2	1.6
AZT/3TC/EFV	1	0.8
Received adherence counseling before ART	n = 122	
Yes	101	82.8
No	21	17.2
Recorded adherence on the chart at the time of taking the viral load	n = 55	
Good	43	78.2
Poor	12	21.8
Missed appointments in the last 1 year	n = 122	
Yes	65	53.7
No	57	46.3
Clinical failure in the last 12 months	n = 54	
Yes	5	9.3
No	49	90.7
Immunological failure	n = 54	
Yes	23	42.6
No	31	57.4
Duration on ART	n = 122	
Less than 3 years	40	32.8
3–5 years	39	32.0
More than 5 years	43	35.2

Eighty-three percent (83%) of the mothers (n = 122) received adherence counselling before initiation on ART including the common side effects of ARV drugs while they received the drugs. Close to 54% of mothers had missed appointments in the last 6 months. Only 78% of the 55 mothers whose adherence charts were accessed at the time of the interview recorded good adherence. Close to 43% had immunological failure. Nearly three quarters of mothers had taken ARVs for more than 3 years.

### Covariates of low viral suppression

Multivariate analysis examined the association between the different characteristics of mothers and adherence. We used adherence to treatment as the common predictor to low viral suppression, as in the case of studies in other jurisdictions [3]. In this study adherence was operationally defined to mean taking drugs every day as prescribed by the health worker. Results in Table 4 indicate that the covariates of low adherence are: taking other medications in addition to ART, missed appointments in the past 6 months, feels like giving up, and experienced violence in the past 6 months (p ≤ 0.05).

**Table 4:** Covariates of low viral suppression among HIV-positive mothers

Background information	Adherent *n* (%)	Non-adherent *n* (%)	Sig	Adjusted (odds ratios)
Mother is taking other medication alongside ARVs				
Yes^a^	12 (52.2)	11 (47.8)	0.000	1.000
No	70 (70.7)	29 (29.3)		6.044
Received adherence counseling before ART				
Yes^a^	71 (70.3)	30 (29.7)	0.527	1.000
No	11 (52.4)	10 (47.6)		0.790
Mothers feels was adequately prepared to start ART				
Yes^a^	63 (71.6)	25 (28.4)	0.178	1.000
No	19 (55.9)	15 (44.1)		0.572
Missed appointments in the last 6 months				
Yes^a^	35 (53.8)	30 (46.2)	0.001	1.000
No	47 (82.5)	10 (17.5)		2.707
Treated differently since disclosing HIV status				
Yes^a^	25 (58.1)	18 (41.9)	0.201	1.000
No	57 (73.1)	21 (26.9)		1.287
There are instances when mother feels like giving up with ARVs				
Yes^a^	12 (50)	12 (50)	0.030	1.000
No	70 (71.4)	28 (28.6)		4.867
Has someone to share with private concerns				
Yes^a^	65 (71.4)	26 (28.6)	0.90	1.000
No	16 (53.3)	14 (46.7)		0.627
Experience violence in the last 6 months				
Yes^a^	34 (54.0)	29 (46.0)	0.053	1.000
No	48 (81.4)	11 (18.6)		2.931
Mother has obstacles to treatment				
Yes^a^	48 (59.3)	33 (40.7)	0.11	1.000
No	34 (82.9)	7 (17.1)		0.299

^a^ Reference category

Specifically, HIV-positive mothers who were not taking other medications were six times more likely to adhere to treatment than those who were taking other medication alongside ARVs (p < 0.001). We further found out that HIV-positive mothers who had missed their appointments in the past 6 months were two times more likely not to adhere to treatment than those who had not missed their appointment (p = 0.001). Mothers who felt like giving up on taking ARVs were almost five times more likely not to adhere to treatment than those who stated expressed no desire to stop medication. Similarly, mothers who experienced some form of violence in the last 6 months were three times more likely not to adhere to treatment than those who never experienced violence.

### Provider perspectives

The results of the focus group discussions with health care providers and linkage facilitators complemented the quantitative survey of HIV-positive mothers and shed further light on its findings. The thematic analysis suggested a range of factors which negatively influenced adherence to ART and put low viral suppression into perspective.

### Health care provider description of mothers with low viral suppression

The characteristics of mothers with low viral suppression was one of the most discussed themes under the study. FGD participants mainly described them as; illiterate, with poor economic backgrounds and who survive on selling food stuffs, lacking support of their husbands, in unstable relationships and having multiple sexual partners and/or marriages and therefore hardly reside in one place, having many children, inconsistently taking their medication because of lack of food or drug stock-out, sharing antiretroviral drugs with husbands or peers, having not disclosed their HIV status to their partners and therefore discreetly taking drugs or not at all, never coming to the facility for scheduled appointments, experiencing different forms of torture and violence, living far away from the health facilities, and mainly in late adolescence.

These comments resonate with the social demographic characteristics described in Table 1 and can be validated by one or more of the following quotations of the FGD participants:

*“Some [of the mothers] don’t have real husbands. They are up and down. That’s why you will not get them easily because they are not in one place*,” FGD participant, Nwoya.

*“Most of them [mothers] lack support of their partners, most of them are not educated and earn a living by selling food stuff that they grow,”* FGD participant, Nwoya.

*“Many of the newly initiated mothers [on ART] have disclosure problems and sometimes don’t disclose their [HIV] status to their spouses,”* FGD participant, Kitgum.

*“These are mothers who have not disclosed their [HIV positive] status to their husbands. They are mothers whose husbands are not aware that their wives are taking drugs…,”* FGD participant, Lira

*“Mothers come from poor families. They lack transport and move on foot. They take drugs on empty stomach. There is gender based violence, [and when] husband chases the woman away, [and] they never come back for refill. Some would [could] stay 2* *months without refill,” *FGD participant, Gulu.

### Health care provider adherence assessment

The practice of assessing adherence had mixed responses. Some providers reported making use of appointment dates or pill count if the mother comes with a bottle, while others depended on self-recall by the mother on the number pills left. In some cases, providers reported to use both methods concurrently. However, it was also common for health providers to qualify their statements by adding that the heavy workload and long queues made it difficult for them to count pills as a way of assessing adherence.

At a FGD in Awach, all providers agreed that the adherence rating on record in the registers was not accurate because it was not based on real adherence analysis but rather on the provider’s quick guess for purposes of recording. This was not so different with other providers elsewhere who mostly attributed this to the workload on any particular clinic day. There was mention that adherence cards which were used to assess adherence in the past are now stocked out. The following quotations support these findings.

*“We ask mothers to come with the remaining pills, but majority do not come with their pills and in this case, we use appointment dates,’’ *FGD participant, Lira.

*“The good or fair assessment of adherence in registers is not real because we don’t delve into pill count because of the large numbers [of clients]…,’’* FGD participant, Gulu.

*“From the ART card there is that column for adherence where if a mother comes for refill we normally ask them looking at the appointment. Then we ask them of the number of the pills they have swallowed,’’ *FGD participant, Apac.

### Health care provider experiences with adherence

Health care providers commonly shared, on the basis of their own experience, that mothers miss their appointments and therefore run out of medicine, they share drugs between themselves and/or with their husbands when either party is unable to come to the facility, and that they stop taking drugs whenever they get new unsuspecting partners. Participants often cited various forms of distress including violence from husbands as prohibitive to mothers taking drugs. It was common for health care providers to state that mothers miss taking drugs especially when they have travelled away, while others think they have recovered from ill health and need not take drugs anymore. The following quotations represent the most common sentiments of providers with regard to mothers’ adherence.

*“I have one mother who told me that her husband does not allow her to take her medicine. So she hides and whenever the man discovers that she has the drugs he throws it in the pit latrine. And right now she has shifted to her mother’s place,’’ *FGD participant, Apac.

*“Some mothers think they are healthy. So [they see] no need to take ARVs. When they get psychological reconstruction, they feel they are healthy,’’* FGD participant, Gulu.

*“Poor adherence [is a result of] some of the women combine [combining] drugs for morning and evening and take [taking] them once in the evening,’’ *FGD participant, Kitgum.

### Health care provider perspectives on non-viral suppression

A key area of exploration in the qualitative component of the study was to obtain providers’ perspectives about why mothers were not suppressing. Common reasons why the respondents believed mothers don’t suppress included: missing appointments and dropping out of care for some time without close monitoring, taking drugs erratically on the account of recovery and good health, torture and violence from their partners, or over time, failing to respond well to the medication. Long distances to the health facility and lack of transport also keep mothers away from care. The shortage of food affects drug use, as mothers will not take drugs on an empty stomach. Similarly, negative perceptions about ARVs, mothers experiencing side effects and stopping taking the drugs, or using them occasionally as and when they feel unwell, and stigma and discrimination keep clients in self-denial and affect adherence and appointments. Representative comments from providers in this regard:

*“…there’s one who stays in [the] village, [and doesn’t follow appointment. She feels cured when she feels her body is fine. That’s what she always says,’’* FGD participant, Apac.

*“Most of them don’t have supportive husbands. They [husbands] want to take their drugs [away from them claiming that]. …..That this transport you are wasting you can use for paying [school fees for] children in school after all you will still die even if you swallow the medicine,’’* FGD participant, Apac.

*“I have a client who kept piling her drugs because [she] believed that the drug will [would] kill her,’’* FGD participant, Lira.

*“Other mothers mistake Septrin for ARVs such that they are confidently swallowing cotrimoxazole in place of ARVs e.g. [we see this] when they run out of stock,’’* FGD participant, Lira.

*“…Pill burden, especially for those with other OIs [opportunistic infections] like TB,’’* FGD participant, Kitgum.

### Provider’s perspectives on early retention

We inquired from health care providers why mothers drop out of care just 3 months after initiation on ART. They identified as contributing factors, that mothers lack proper identifying information for follow-up and non-disclosure to their partners for fear of reprimand, violence or separation. The following quotes provide an account of this phenomenon:

*“They use common names in villages like maama John, and follow*-*up is not easy [with such names],’’* FGD participant, Gulu.

*“Disclosure to husband will cause divorce and cause mother [to] move back [be chased away] to her parents. So she keeps quiet and disappears [from routine care],’’* FGD participant, Apac.

Results of the study among HIV-positive mothers revealed that 17% of the 122 mothers never received adherence counseling before initiation on ART. This was confirmed by providers as follows:

*“Sometimes midwives counsel them, but not to [their] expectation, especially that the [HIV] positive person needs a lot of time,’’ *FGD participant, Gulu.

*“It’s the pregnancy that will lead them [to] know their status and once they know, it’s shocking and [they end up] leave [leaving] the facility. HIV status is detected only during ANC so it’s a shocking moment,’’* FGD participant, Gulu.

*“Some mothers will enter denial stage and go to another health facility. So one client can be registered twice, [and this is] common in town,’’* FGD participant, Kitgum.

*“[They have for the] Disbelief of the results and [they] will deny the results and they will go away….,’’ *FGD participant, Gulu.

## Discussion of results

This study collected data on different variables from HIV-positive mothers with known low viral suppression only, since looking at these characteristics in all HIV-positive women would bias results; given that their characteristics may not be similar or have the same distribution in the women with good viral suppression.

Unfortunately, the rate of adherence in our sample was significantly lower (67.2%) than has been observed in many other studies in Africa, even when it was self-reported adherence. For example, a study on adherence and low viral response in Kisumu, Kenya found adherence at 87.1% among women on Option B+ [6]. Many factors identified in this study would explain this problem. While socio-economic factors in this study are consistent with other literature in influencing adherence, the majority of our sample comprised a relatively young population with unique characteristics.

Up to 23% of women in this study had multiple sexual partners. This was corroborated by health care providers that most women who have not achieved the required suppression have unstable relationships and were less likely to disclose their HIV status to their non-regular partners for fear of breakdown of their relationship. They were more prone to violence and vulnerable to discrimination by others even before the effect of other socio-economic conditions that generally affect women on ARVs. These could be termed as frontline factors, both of which could be used to explain this finding and were corroborated by the health care providers.

The cultural context in Northern Uganda, a post-conflict region, is such that women are sexual subordinates and most often responsible for care of the household and children, and as a consequence, these practical barriers may adversely affect adherence among women. Perhaps, these among other factors, make the sample not comparable with women in other studies whose socio-economic variables could have different levels of influence on treatment adherence.

Another key adherence barrier raised during the interviews included violence from their partners. Our findings reinforce the importance of partner counseling, including obtaining remedies for victims of violence whenever they exceed the confines of counseling.

Adherence counseling prior to initiation of ART was associated with consistency in taking drugs. It was further discovered that mothers who had missed appointments in the last 6 months were more than two times less likely to adhere to their treatment than HIV-positive mothers who never missed their appointments at all. Adherence counseling before ART is one way to determine readiness to start treatment, diagnosing and addressing stigma, depression, and initiating a patient-provider relationship to even understand the patient better [7]. Such a missed opportunity has long-term effects that would not be reversed when clients slowly become complacent to treatment.

Even HIV-positive mothers who were treated discriminately since disclosure of their HIV status were only slightly less likely to take their drugs consistently as compared to those who were not discriminated/stigmatized. It is possible that patients with limited literacy might be reluctant to ask others for the kind of help they need to take their medicines correctly. Better-educated patients who are convinced of ART efficacy, perhaps as a result of educational programs, show a propensity towards better adherence [8]. Therefore, adherence interventions among our sample which was predominantly illiterate need to consider these factors when reinforcing continuing adherence practices. It may be necessary to schedule follow-ups that are more frequent, monitor adherence more closely, and ensure that education and discussions take place in a safe environment.

Patients’ beliefs, knowledge, and expectations regarding treatment strongly influence medical decision making [9]. Our findings from both mothers and health care providers showed that a few mothers believed that ARVs kill, are like any other medication such as Panadol, or have the same effect as cotrimoxazole prophylaxis or even think that one can take substitute ARVs with Septrin. Therefore, misconceptions towards the disease and treatment regimens could explain the poor therapeutic outcomes that in turn may impede achievement of optimal levels of adherence. However, this argument is limited by the fact that this study did not ascertain whether the mothers had received health education on PMTCT, an important aspect that could have influenced mother’s beliefs, knowledge and expectations regarding treatment as well as adherence to ART.

Although ART is free of charge throughout Uganda’s public health system, there are ART-associated costs such as expenses in transport and food. These obstacles were cited by mothers and corroborated by health care providers. Mothers cited skipping medication because of lack of food or went without medication because they could not reach the health facility. This is consistent with other studies which have linked costs with poor adherence to treatment [6].

A study in Botswana showed that adherence was higher when cost was not an obstacle and that poor patients were able to achieve excellent rates of adherence when they had access to free laboratory monitoring and subsidized ART [10]. This is a useful finding, and it is important that policy makers use this information to introduce outreach programs for ART to cut key costs associated with transport.

In the FGDs, participants raised the issue that mothers were worried of disclosing their status to partners for fear of losing them. As such they either take drugs in hiding or cease taking them completely. Like many other studies, non-disclosure of HIV status was associated with poorer adherence. Our findings suggest unless mothers disclose, they cannot effectively take their drugs. This could perhaps explain the high rates of missed appointments (53.7%). This is a fundamental issue that disclosure health education and individual counselling sessions should embrace with utmost importance.

Distance to health facilities was a common cause of missed appointments, as was drug stock-out as reported by mothers and validated by health care providers in the FGDs. Over half of the mothers come as far as 10 or more kilometers to the health facilities and find the transport cost prohibitive. This coupled with the seasonal deterioration of poorer roads during the rainy season makes it even harder to access facilities. Deliberate outreaches in hard-to-reach areas would offset this limitation as much as possible.

ART regimens have toxicities and adverse side effects that vary from mild to severe and acute to chronic, which can prevent adherence [11]. Our study revealed that both mothers and health care providers cited side effects as one of the key barriers to taking medication promptly. This is worsened by the scarcity of food to support treatment. Adherence counseling together with follow-up and clinical support for mothers, especially at early initiation onto ART, would help guide mothers on how to cope with side effects. These may be tackled by informing patients in advance what side effects to expect and how they might be managed, as well as prescribing antiemetic or anti-diarrheal agents to alleviate these side effects.

Food was an important barrier to medication in this study. Previous research has shown a relationship between food insecurity (uncertain access to safe and nutritious food) and lower rates of viral suppression among ART-treated patients in the US and Uganda. Pregnant and breastfeeding women may be especially vulnerable to food insecurity and insufficiency [12].

## Conclusions

Adherence of 67.2% among mothers with low viral suppression in this study is very low. The main covariates were distance to travel to health facility leading to missed appointments and running out of drugs, sharing antiretroviral drugs, drug side effects, lack of pre-ART counseling, gender-based violence, social lifestyles such as multiple sexual partners, non-disclosure to partners, and stigma and discrimination. Qualitative findings from providers corroborated these findings. To improve the quality of care provided to mothers, the information from this study can be used to screen pregnant women and lactating mothers on ART for any of the factors strongly associated with low adherence to ART (and thus low viral load suppression) and then provide support through intensive adherence counselling and self-management or peer-to-peer support.

## Abbreviations

ART: antiretroviral therapy
ARVs: antiretroviral drugs
AIDS: acquired immune deficiency syndrome
ASSIST: Applying Science to Strengthen and Improve Systems
AZT: zidovudine
CDC: Centers of Disease Control and Prevention
CD4: cluster of differentiation 4-positive cell
CI: confidence interval
EFV: efavirenz
EPIDATA: epidemiological data version 3
EMTCT: elimination of mother-to-child transmission
EID: early infant diagnosis
FGD: focus group discussion
HAART: highly active antiretroviral therapy
HIV: human immunodeficiency virus
HC: Health Center
HCT: HIV counseling and testing
3TC: lamivudine
MTCT: mother-to-child transmission
MOH: Ministry of Health
NVP: nevirapine
OR: odds ratio
PEPFAR: U.S. President’s Emergency Plan for AIDS Relief
PLHIV: people living with HIV/AIDS
PMTCT: prevention of mother-to-child transmission
TDF: tenofovir
UNAIDS: Joint United Nations Programme on HIV/AIDS
USAID: United States Agency for International Development
